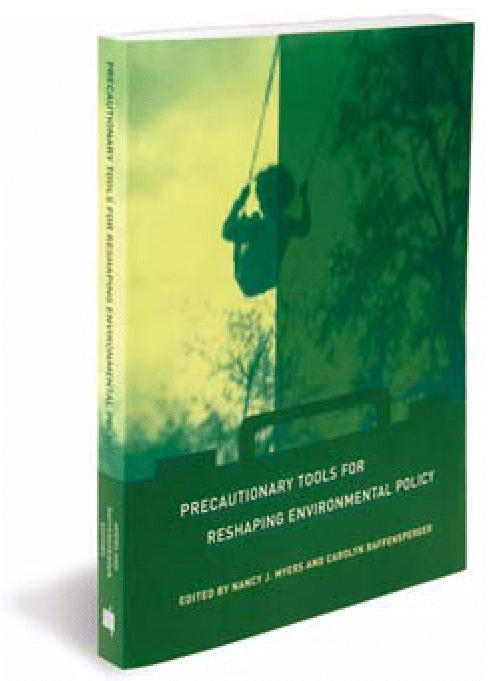# Precautionary Tools for Reshaping Environmental Policy

**Published:** 2006-04

**Authors:** Peter Saunders

**Affiliations:** Peter Saunders is Professor Emeritus of Mathematics at King’s College London, UK, co-founder of the Institute of Science in Society, and a member of the Independent Science Panel. He has written several articles on the precautionary principle and is an associate editor of Science in Society.

Edited by Nancy J. Myers and Carolyn Raffensberger

Cambridge, MA:MIT Press, 2006. 400 pp. ISBN: 0-262-13458-6, $62 cloth; ISBN: 0-262-63323-X, $25 paper.

Opponents of the precautionary principle often complain that it is not a sufficient tool for decision making. They’re right—except that, as far as I know, no one ever said it was. It should be part of the process, but it can be only a part, and Myers and Raffensberger have put together an excellent guidebook for those who want to implement it.

Perhaps they’ll even convince some who still doubt its usefulness. The volume begins with a checklist of points that we should consider when decisions are being made. Nothing dramatically new—all good common sense, really—but the ideas are important and too often overlooked. The contributors then expand on the points and give examples of how they have—or have not—been put into practice. These illustrations are valuable intrinsically; also, when you are trying to convince your government to act, it helps to have examples of how other countries have taken similar action without dire economic or social consequences, and how doing nothing may turn out to be more expensive in the long run, or even the not-so-long run.

Anyone interested in environmental policy will find this book stimulating and useful, but the editors themselves acknowledge that there is much more to be done, and in my view what is most needed is more input from scientists. As far as I can tell, although some of the contributors were trained in science, none is currently employed as a scientist. That’s not really surprising in a book about policy, but working scientists do have a different perspective on things.

People who are not scientists often place too much credence in what the corporations and the scientific establishment tell them. It is certainly important to understand that the arguments are about values as well as science, but that doesn’t mean we should let the science-based points go by default. Just as we demand that our opponents take values into account, we must fully understand the scientific arguments. And there is no more effective way of making your case than to show that your opponents are wrong even on their own terms—as they often are.

Despite what you’ve been told about the uncertainty that is inherent in science, many scientists are not above making unjustifiably sweeping claims, especially when writing for laypeople. In particular, you should always be suspicious of any statement that begins “There is no evidence for …” You often hear this said about the possibility that genetically modified (GM) organisms can be harmful. Yet there is no shortage of evidence that they can: rats fed on GM potatoes, mice fed on GM peas (especially worrying because the protein involved is harmless in the bean from which the gene was transferred), and, most alarming of all, humans in the Philippines who were affected by pollen from GM maize.

If there seems to be no evidence, that may be only because no one has ever looked for any. The sort of research that is needed is often unattractive to scientists, not least because it can make them unpopular with many of their colleagues and with the big corporations, and so hinder their careers. The case of Quist and Chapela’s finding of transgenes in GM maize, described in the book, is just one example among many that have been reported, and we have no way of knowing how many others there have been that never came to light. There is also, of course, the simple fact that it costs money to do research, and work that does not suit the people who control the funding tends not to get done.

So I would add to the checklist that we should not take for granted what the other side says about the science. If they tell you there is no evidence for something, ask what experiments, if any, have been carried out to look for it. If you are not satisfied with the answer, get on the web and see what you can find. You don’t have to be able to debate the issues with the experts; simply raising a problem that they would rather ignore may be enough to alert the regulators. There was a lot more official concern about horizontal gene transmission in the United Kingdom after it was discussed in an episode of *The Archers*, a popular radio soap.

In the meantime, I’ll keep my copy of this volume where I can reach it.

## Figures and Tables

**Figure:**